# Delta radiomics: an updated systematic review

**DOI:** 10.1007/s11547-024-01853-4

**Published:** 2024-07-17

**Authors:** Valerio Nardone, Alfonso Reginelli, Dino Rubini, Federico Gagliardi, Sara Del Tufo, Maria Paola Belfiore, Luca Boldrini, Isacco Desideri, Salvatore Cappabianca

**Affiliations:** 1 Department of Precision Medicine, University of Campania “L. Vanvitelli”, 80138 Naples, Italy; 2 Dipartimento di Diagnostica per Immagini, Radioterapia Oncologica ed Ematologia, Fondazione Policlinico Universitario “A. Gemelli” IRCCS, Rome, Italy; 3 Department of Biomedical, Experimental and Clinical Sciences “M. Serio”, University of Florence, Florence, Italy

**Keywords:** Delta radiomics, Radiomics, Texture analysis, Meta-analysis, Oncology, Precision medicine, Radiotherapy

## Abstract

**Background:**

Radiomics can provide quantitative features from medical imaging that can be correlated with various biological features and diverse clinical endpoints. Delta radiomics, on the other hand, consists in the analysis of feature variation at different acquisition time points, usually before and after therapy. The aim of this study was to provide a systematic review of the different delta radiomics approaches.

**Methods:**

Eligible articles were searched in Embase, Pubmed, and ScienceDirect using a search string that included free text and/or Medical Subject Headings (MeSH) with 3 key search terms: 'radiomics,' 'texture,' and 'delta.' Studies were analyzed using QUADAS-2 and the RQS tool.

**Results:**

Forty-eight studies were finally included. The studies were divided into preclinical/methodological (5 studies, 10.4%); rectal cancer (6 studies, 12.5%); lung cancer (12 studies, 25%); sarcoma (5 studies, 10.4%); prostate cancer (3 studies, 6.3%), head and neck cancer (6 studies, 12.5%); gastrointestinal malignancies excluding rectum (7 studies, 14.6%) and other disease sites (4 studies, 8.3%). The median RQS of all studies was 25% (mean 21% ± 12%), with 13 studies (30.2%) achieving a quality score < 10% and 22 studies (51.2%) < 25%.

**Conclusions:**

Delta radiomics shows potential benefit for several clinical endpoints in oncology, such asdifferential diagnosis, prognosis and prediction of treatment response, evaluation of side effects. Nevertheless, the studies included in this systematic review suffer from the bias of overall low methodological rigor, so that the conclusions are currently heterogeneous, not robust and hardly replicable.

Further research with prospective and multicenter studies is needed for the clinical validation of delta radiomics approaches.

**Supplementary Information:**

The online version contains supplementary material available at 10.1007/s11547-024-01853-4.

## Introduction

Radiomics, the extraction of quantitative imaging features from medical images, has proven valuable in developing models for cancer diagnosis, patient prognosis, and clinical decision support [1–6]. A recent evolution in radiomics, termed "delta texture analysis" or "delta radiomics," has emerged to account for feature variations at different acquisition time points. This novel approach enables the examination of feature changes following specific steps in the patient's workflow, such as therapy, timing, or biological events [7, 8]. The potential of delta radiomics lies in its ability to offer insights into the effects of interventions and guide adaptive treatment strategies based on its predictive capabilities [9–11].

Despite the promise of delta radiomics, existing studies have yielded conflicting and heterogeneous results, impeding the generalizability and applicability of this approach [12, 13]. Therefore, the present study aims to conduct an updated and comprehensive meta-analysis, incorporating the latest literature available up to December 2024, to reevaluate delta radiomics and scrutinize the quality of the studies using contemporary radiomics research evaluation criteria [13]. Additionally, we will perform a comparative analysis between the findings of the previous meta-analysis (up to August 2021) and the new literature to identify trends, disparities, and advancements in the field. We will also examine ongoing trials identified in the clinicaltrials.gov registry.

## Methods

Adhering to the PRISMA statements [14], our systematic review encompassed a thorough literature search, focusing on English articles registered in Embase, PubMed, and ScienceDirect. Unlike the previous analysis, we did not set a specific start date, and the search results were last updated in December 2023. The search string incorporated free text and/or Medical Subject Headings (MeSH) with the primary terms 'radiomics,' 'texture,' and 'delta.' Duplicate results were removed, and all references cited in the retrieved results were meticulously examined.

Inclusion and exclusion criteria mirrored the previous analysis, with eligibility criteria comprising English original articles focusing on temporal studies of delta radiomics. Conversely, exclusion criteria included case reports, review articles, studies investigating spatial delta radiomics approaches, and poster presentations or conference abstracts. Three reviewers (FG, DR and SDT) independently screened all articles, and discrepancies were resolved through panel discussions with two additional authors (VN and AR). The same search string was used to identify prospective trials in the clinicaltrials.gov registry.

### Quality assessment and data analysis

To assess the quality of the selected studies, two reviewers (VN and GV) independently employed the Radiomics Quality Score (RQS) [2] and the Quality Assessment of Diagnostic Accuracy Studies-2 (QUADAS-2) [15] tools. The QUADAS-2 aimed to quantify diagnostic accuracy, while the RQS tool summarized validity and potential bias in radiomics studies, with a maximum score of 36 points. Results were reported as a percentage of the maximum score (x/36%). Any disagreements between reviewers were resolved through consensus discussions. Subsequently, a comparative analysis was conducted to discern patterns and variations between the findings of the previous literature and the new literature.

## Results

Between August 16, 2021, and December 31, 2023, a total of 460 records were initially identified. Following the exclusion of duplicate and irrelevant titles and abstracts, 387 studies were excluded. The remaining records underwent a more detailed analysis. Only two additional references were included after a thorough evaluation of the reference lists of the selected articles. In contrast, 17 articles were excluded—nine studies because they did not focus on temporal delta radiomics and eight studies as they were not original articles.

Consequently, a total of 58 studies were incorporated into this systematic review.

**Fig. 1:**
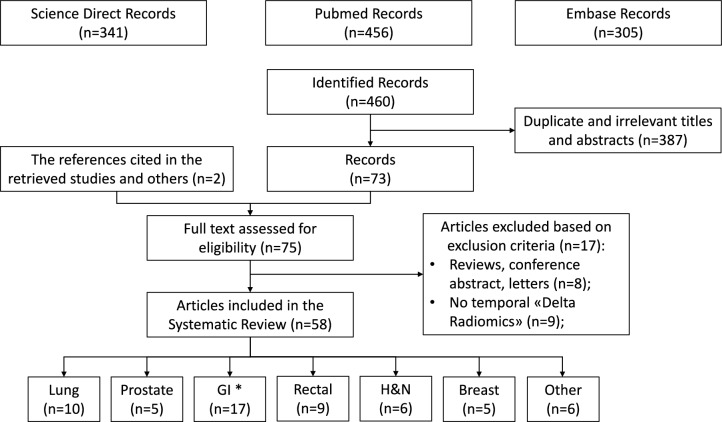
Flowchart of the study. GI: gastrointestinal cancer. HN: head and neck cancer. * GI excludes rectal cancer that are treated in another paragraph

To simplify presentation, we categorized the 58 included studies into distinct clinical groups: non-rectal gastrointestinal disease (17 studies, 29.3%), lung cancer (10 studies, 17.2%), rectal cancer (9 studies, 15.5%), head and neck cancers (6 studies, 10.3%), breast cancer (5 studies, 8.6%), prostate cancer (5 studies, 8.6%), and other disease areas (6 studies, 10.3%). The flowchart illustrating the study selection process is depicted in Fig. 1.

Most of the selected studies were retrospective (49 studies out of the 58 clinical trials: 85%), with only 9 prospective clinical trials published (15%). The most common imaging modality was MRI (31 studies, 53%), followed by CT (16 studies, 27%), PET/CT (5 studies, 8%), CBCT (4 studies, 6%) and ultrasonography (2 studies, 3%).

### Quality analysis of the included studies

**Fig. 2:**
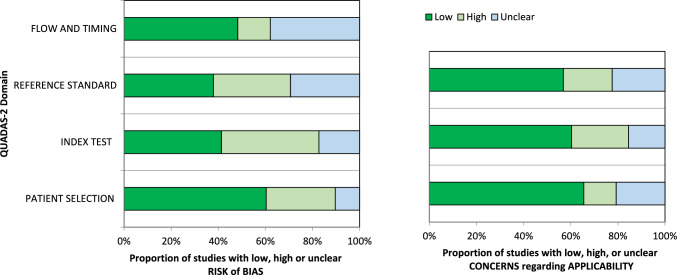
Grouped bar graphs of risk of bias and applicability concerns for the included diagnosis-related studies using QUADAS-2

Assessment of the quality of the 58 included studies was conducted using QUADAS-2, and the results are presented in Fig. 2. In terms of patient selection, 23 studies (39.6%) were identified with a high or unclear risk of bias, primarily due to the ambiguity in their inclusion criteria.

Concerning the index test, 34 studies (58.6%) were categorized as high or unclear risk, due to the absence of a defined threshold and the lack of blinding between the reference standard and the index risk itself. Risk of bias related to the reference standard, flow, and timing was determined to be high or unclear in 36 (62.1%) and 30 (51.7%) studies, respectively, owing to uncertainties surrounding the reference standard.

The applicability of QUADAS-2 was also deemed unclear or highly questionable in the areas of reference standard (20 studies, 34.5%), index test (23 studies, 49.1%), and patient selection (25 studies, 43.1%) (refer to Fig. 2). It is noteworthy to emphasize that these scores, while raising concerns, demonstrate an improvement compared to the results of the recent previous meta-analysis (refer to Supplementary).

**Fig. 3:**
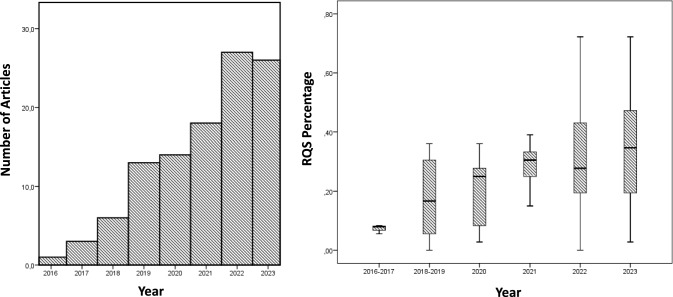
Boxplot of RQS percentage of clinical trials included in the meta-analysis by year of publication

The median RQS of all studies was 30.5% (mean 31% ± 17%), with 6 studies (10.3%) having a quality score below 10% and 21 studies (36.2%) below 25%. Notably, there has been a consistent trend in recent years toward an increase in the RQS score of included studies, as illustrated in Fig. 3. Eighteen clinical trials did not perform any type of validation (31%), while 29 trials performed internal validation (50%) and 11 trials performed external validation (19%).

Table 1 summarizes the characteristics of the clinical trials included in the present meta-analysis.

**Table 1:** Clinical studies analyzed in the present meta-analysis

Authors	Year	Study type	Setting	Number pts	Imaging	Endpoint	Validation
Xiumei Li	2023	Lung	Retrospective	96	CT	Clinical (acquired response to EGFR-TKIs)	Internal validation
Francois Cousin	2023	Lung	Retrospective	188	CT	Clinical (response prediction to immune checkpoint inhibitors)	Internal validation
Olena Tankyevch	2022	Lung	Retrospective	83	PET\TC	Clinical (response prediction to IT)	External validation
Yingpu Cui	2022	Lung	Retrospective	29	PET\TC	Clinical (response prediction after neoadjuvant IT with chemotherapy)	External validation
Dong Xie	2022	Lung	Retrospective	97	CT	Clinical (response prediction to IT)	Internal validation
Yanquing Ma	2022	Lung	Retrospective	1094	CT	Clinical (response prediction in MPLA and SPLA)	No validation
Marco Bertolini	2022	Lung	Retrospective	117	PET\TC	Clinical (response prediction SBRT in NSCLC)	External validation
Emanuele Barbarino	2022	Lung	Retrospective	33	CT	Clinical (response prediction to IT)	No validation
Ruiping Zhang	2022	Lung	Retrospective	10	CT	Clinical (response prediction)	No validation
Parisa Forouzannezhad	2022	Lung	Retrospective	45	CT	Clinical (prediction of survival outcomes after RTCT stage IIb-IIIb NSCLC)	Internal validation
Abhishek Midya	2023	Prostate	Retrospective	121	MRI	Clinical (prediction of progression during active surveillane)	External validation
Nikita Sushentsev	2023	Prostate	Retrospective	76	MRI	Clinical (prediction of progression during active surveillane)	External validation
Rodrigo Delgadillo	2022	Prostate	Retrospective	50	CBCT	Clinical (prediction of genitourinary toxicities)	Internal validation
Ahmad Algohary	2022	Prostate	Retrospective	25	MRI	Clinical (response prediction after LEAD boost RT)	No validation
Nikita Sushentsev	2022	Prostate	Retrospective	64	MRI	Clinical (prediction of progression during active surveillane)	External validation
Lei Miao	2023	Sarcoma	Retrospective	30	MRI	Clinical (response prediction RT + Target therapy)	No validation validation
Xi	2022	H&N	Retrospective	96	MRI	Clinical (CRT response prediction)	Internal validation
Corino	2022	H&N	Retrospective	50	MRI	Clinical (CRT response prediction)	Internal validation
Sellami	2021	H&N	Retrospective	93	CBCT	Clinical (CRT progression prediction)	Internal validation
Morgan	2021	H&N	Retrospective	90	CBCT	Clinical (local failure prediction)	Internal validation
Abdollahi	2023	H&N	Retrospective	31	CBCT	Clinical (xerostomia prediction)	Internal validation
Kim	2023	H&N	Retrospective	145	PET	Clinical (Correlation with Survival)	No validation
Liu	2023	Breast	Prospective	120	MRI	Clinical (response to NAC)	Internal validation
Guo	2022	Breast	Prospective	140	MRI	Clinical (response to NAC)	Internal validation
Bhardwaj	2022	Breast	Prospective	83	US	Clinical ( local failure prediction)	Internal validation
Yang	2022	Breast	Retrospective	217	US	Clinical (Correlation with Ki67 status)	Internal validation
Khorrami	2023	Breast	Retrospective	73	CT	Clinical (response prediction)	Internal validation
Small	2023	Cervical	Retrospective	39	MRI	Clinical (PFS prediction)	No validation
Esposito	2023	Lymphoma	Retrospective	79	PET	Clinical (PFS prediction)	No validation
Guerrisi	2021	Melanoma	Retrospective	32	CT	Clinical (PFS prediction)	Internal validation
Li	2022	Moyamoya	Retrospective	53	CT	Clinical (recurrence prediction)	Internal validation
Gerwing	2022	Venous Malformation	Prospective	16	MRI	Clinical (outcome prediction)	No validation
Wen	2023	Rectal cancer	Retrospective	126	MRI	Clinical (TRG after nCRT)	Internal validation
Chiloiro	2022	Rectal cancer	Prospective	43	MRI	Diagnostic (complete response and organ preservation)	Internal validation
Chiloiro	2023	Rectal cancer	Retrospective	203	MRI	Diagnostic (mesorectal radiomic features for a complete response)	Internal validation
Chiloiro	2022	Rectal cancer	Retrospective	48	MRI	Diagnostic (results after nCRT)	External validation
Chiloiro	2023	Rectal cancer	Prospective	63	MRI	Diagnostic (ERI for predicting a complete responce in LARC)	No validation
Wang	2023	Rectal cancer	Retrospective	84	MRI	Diagnostic (predicting treatmente response in LARC after nCRT)	External validation
Nardone	2022	Rectal cancer	Retrospective	37	MRI	Diagnostic (response il LARC addressed to nCRT and surgery)	External validation
Peng	2023	Rectal cancer	Retrospective	83	MRI	Clinical (pCR in patients undergoing neadjuvant treatment)	No validation
Fang	2023	Rectal cancer	Retrospective	83	MRI	Clinical (predicting post-nCRT lymph node status in patients with LARC	Internal validation
Li	2023	GI	Retrospective	95	CT	Clinical (pCR in ESCC treated with neoadjuvant IT, CT and surgery)	Internal validation
Kriashna	2023	GI	Retrospective	166	CT	Clinical (survival outcames in metastatic GEAs)	No validation
Lu	2023	GI	Prospective	108	MRI	Diagnostic (pCR in ESCC after neoadjuvant CT)	Internal validation
An	2022	GI	Prospective	76	MRI	Diagnostic (response prediction in ESCC treated with cCRT)	Internal validation
Shen	2023	GI	Retrospective	177	CT	Clinical (predict long-term efficacy after neoadjuvant CT in ADC)	External validation
Li	2023	GI	Retrospective	42	CT	Clinical (predicting the prognosis of patients with stage IV gastric cancer treated with ICI)	No validation
Wang	2023	GI	Retrospective	103	CT	Diagnostic (TRG for gastric cancer treated with neoadjuvant chemotherapy)	Internal validation
Aujay	2022	Liver	Retrospective	14	MRI	Diagnostic (TARE) in patients with locally advanced HCC)	No validation
Ho	2023	Liver	Retrospective	26	MRI	Diagnostic (treatment response to a novel sequential TACE plus SBRT plus immunotherapy regimen in unresectable HCC)	No validation
Jin	2022	Liver	Retrospective	22	MRI	Diagnostic (prediction of local control in liver lesion trated with SBRT)	No validation
Han	2023	Liver	Retrospective	126	MRI	Clinical (value of multi-phase contrast-enhanced magnetic resonance imaging based on the delta radiomics model for identifying glypican-3 (GPC3)-positive HCC)	Internal validation
Ye	2022	Liver metastases	Retrospective	139	CT	Clinical (predict the therapeutic efficacy and PFS of patients with liver metastasis of colorectal cancer)	Internal validation
Xuan	2023	Liver metastases	Retrospective	100	MRI	Diagnostic (PFS in patients with colorectal liver metastases undergo CT)	Internal validation
Giannini	2022	Liver metastases	Retrospective	242	MRI	Diagnostic (efficacy and outcomes after treatment of colorectal liver metastases)	External validation
Ma	2021	Liver metastases	Retrospective	102	MRI	Diagnostic ( chemotherapy response in synchronous liver metastasis)	Internal validation
Simpson	2022	Pancreas	Retrospective	30	MRI	Diagnostic (predicting response of pancreatic cancer patients treated with magnetic resonance image guided SBRT)	No validation
Tomaszewski	2021	Pancreas	Retrospective	26	MRI	Diagnostic (MRgRT for treatment response prediction)	No validation

Abbreviations: LEAD: Lattice extreme ablative dose, IT: Immuno therapy, MPLA: Multiple primary lung adenocarcinoma, SPLA: Solitary primary lung adenocarcinoma, EGFR-TKIs: Epidermal growth factor receptor tyrosine kinase inhibitors, ERI: Early tumor regression index, LARC: Locally advanced rectal cancer, pCR: patological complete response, nCRT: neoadjuvant chemoradiation therapy, ESCC: Esophageal squamous cell carcinoma, GEAs: Gastroesophageal adenocarcinomas, cCRT: concurrent chemoradiotherapy, ADC: Advanced gastric cancer, ICI: Immuno checkpoint inibitors, TRG: Tumor regression grade, TARE: Transarterial radioembolization, PFS: Progresson free survival, MRgRT: RT RM-guided, NAC: Neoadjuvant chemotherapy

#### Lung cancer

In this update, ten novel studies on the use of delta radiomics in lung cancer have been found, dealing with different aspects of the disease.

Bertolini et al. investigated the relationship between radiomic features extracted from pre-treatment CT and PET/CT scans and the clinical outcomes of stereotactic body radiotherapy (SBRT) in early-stage NSCLC. Harmonized radiomic models demonstrated the ability to accurately predict patient prognosis [16].

Other authors focused on stage III non small cell lung cancer (NSCLC), in different settings. Cui et al. developed a comprehensive PET/CT radiomic model predicting pathological response after neoadjuvant toripalimab (PD-L1 and PD-L2) with chemotherapy in resectable stage III NSCLC patients [17].

Patients underwent baseline PET/CT, followed by three cycles of neoadjuvant toripalimab with chemotherapy, a PET/CT three weeks after completing neoadjuvant treatment, and finally surgical resection.

Zhang et al. analyzed CT radiomics data to determine at which point, after the start of treatment, radiomics shows the most significant change in stage III NSCLC patients treated with RT or RT-CT, identifying two time points with the highest change rates: week 1 and week 3 [18]. Forouzannezhad et al. through the study of ^18^FDG-PET/CT, CT, and SPECT images, investigated the utility of multitasking multi-temporal radiomic features compared to single-task learning in improving survival outcome prediction compared to conventional clinical imaging feature benchmark models [19].

Several authors investigated the potential use of delta-radiomics in the prediction of clinical outcomes in patients undergoing immune-checkpoint inhibitors. Xie et al. based on delta radiomic data combined with clinical-pathological features, created a model to distinguish between patients with slow and rapid progression to immune checkpoint inhibitors (ICI) treatment [20]. Barabino et al. analyzed lung lesions from CT examinations at baseline and the first reevaluation in NSCLC patients treated with ICI to assess the predictive ability of 27 radiomic features for each lesion [21]. Tankyevych et al. demonstrated the potential of specific radiomic features extracted from initial and follow-up PET/CT scans, along with their evolution, in predicting clinical outcomes, progression, therapy response, overall survival (OS), and progression-free survival (PFS) in NSCLC patients treated with immunotherapy, predicting favorable treatment outcomes [22].

Cousin et al. evaluated the potential role of CT-based radiomics in predicting treatment response and survival in advanced NSCLC patients treated with PD-1/PD-L1 inhibitors, identifying patients who might not have greater benefit [23].

Conversely, Li et al. analyzed follow-up non-contrast-enhanced CT images of lung adenocarcinoma patients to evaluate the predictive value of delta radiomic features in predicting resistance to EGFR-TKIs receptor tyrosine kinase inhibitors [24]

Finally, Ma et al. evaluated the difference between multiple primary lung adenocarcinoma (MPLA) and solitary primary lung adenocarcinoma (SPLA) using machine learning algorithms based on delta radiomics in CT images, and demonstrated higher accuracy with longer follow-up [25].

#### Prostate cancer

In this update, five studies regarding prostate cancer have been included. Different authors have investigated the role of delta-radiomics to refine patients under active surveillance (AS). In this regard, the purpose of the study by Midya et al. was to quantify radiomic variations in the progression of prostate cancer (PCa) using sequential magnetic resonance imaging (MRI) in patients undergoing AS and assess their association with pathological progression on biopsy [26]. Preliminary results suggest that delta radiomics is more strongly associated with upgrade events than PIRADS and other clinical variables. Similarly, Sushentsev et al. focused on MRI images in patients with AS prostate cancer and developed the first predictive model that analyzes the combination of radiomic and clinical features sequentially over time [27].

They enriched the conventional approach of delta radiomics by using a Long Short-Term Memory Recurrent Neural Network (RNN). The same group compared the performance of the PRECISE scoring system with different delta-radiomics models to predict the histopathological progression of prostate cancer [28].

PRECISE and delta-radiomics models achieved comparably good performance in predicting PCa progression in AS patients.

Other authors have investigated the role of delta-radiomics for the prediction of toxicities and outcomes in patients undergoing RT.

Delgadillo et al. focused on genitourinary toxicities in patients with prostate cancer treated with definitive RT, using CBCT to ensure treatment accuracy [29]. They identified a delta model to predict acute and subacute International Prostate Symptom Scores (IPSS) and toxicity grades. Already in the first week of RT, corresponding to the first 20 Gy BED, CBCT-based delta radiomics predicted acute and subacute GU toxicity and ∆IPSS with moderate performance (AUC > 0.7). Conversely, Algohary et al. studied pre-RT and post-RT MR images, in patients with prostate cancer undergoing RT [30]. The objective was to evaluate the predictive capabilities of certain radiomic features regarding treatment outcomes.

#### Gastrointestinal cancers

Delta radiomics has been employed in various studies as a promising and non-invasive method also for gastrointestinal diseases, particularly for the prediction of treatment response and support in the differential diagnosis.

#### Esophageal and gastric *cancer*

Li et al. examined the capability of machine learning models based on delta radiomic features from CT images in patients with squamous cell esophageal carcinoma. By utilizing the variation in image group characteristics before and after immunochemotherapy, they established machine learning models and compared these models with those based solely on post-immunochemotherapy CT images. The aim of their study was to avoid unnecessary surgery in patients responsive to immunotherapy. Their decision curve analysis showed that their machine learning models had good predictive performance and provided reference values for the clinical treatment decision-making process, yielding better results on complete pathological response [31].

Krishna et al. conducted a retrospective study on 166 patients with metastatic gastroesophageal adenocarcinoma undergoing palliative chemotherapy with contrast-enhanced CT. Their study aimed to identify the combination of clinical, radiomic, and delta radiomic features to accurately predict progression-free survival and overall survival. Their study has shown that the number of metastatic lesions after 8–12 weeks of chemotherapy is important in predicting survival. Furthermore, they demonstrated that the presence of brain metastases correlates with poor overall survival (OS) but not progression-free survival (PFS). Among radiomic features, contrast and shape compacity were utilized. The difference in intensity between neighboring regions was identified as a marker of intratumoral heterogeneity and is associated with both PFS and OS. Loss of heterogeneity and improvement in homogeneity on CT before and after treatment are well-known indicators of treatment response, while shape compacity reflects volume compactness. Greater compacity correlates with worse PFS [32].

Lu et al. evaluated the effectiveness of the delta radiomic model in predicting treatment response in patients with locally advanced squamous esophageal carcinoma undergoing neoadjuvant chemotherapy, using MRI studies. The study demonstrated that MRI radiomic features based on T2-TSE-BLADE sequences had the potential ability to predict TRG (tumor regression grade). Magnetic resonance imaging features of the lesions were extracted using the T2-TSE-BLADE image, which is capable of providing high image quality while reducing motion artifacts. Four radiomic features were selected for constructing the Delta model along with the shape feature. It was found that there was no statistically significant difference in volume reduction rates between good responder and non-good responder groups. The most important feature was Gray Level NonUniformity Normalized, exhibiting a lower intensity level in good responder patients [33].

An et al. assessed the association between radiomic features extracted from an ADC map of the entire tumor during early treatment (5th fraction) and after concurrent chemoradiation treatment (10th fraction) in patients with squamous cell esophageal carcinoma. Radiomic features were extracted from pre-treatment images and delta radiomics series, and predictive models were set up. It was demonstrated that delta radiomics based on ADC map during chemoradiation was effective in predicting treatment response [34].

Similarly, studies on delta radiomics have been conducted for gastric tumors. Shen et al. studied 132 patients as an internal cohort and 43 patients as an external validation cohort with advanced gastric carcinoma treated with neoadjuvant chemotherapy. They aimed to evaluate the association between changes in radiomic features on computed tomography before and after treatment and predict overall survival (OS) The LASSO method was used to select predictive radiomic features. The analysis of AUC was performed on three CT parameters: acquisition of radiological features of CT before neoadjuvant chemotherapy (befCT-RS), acquisition of radiological features of CT after neoadjuvant chemotherapy (aftCT-RS), and variations in the values of acquisition of radiological features of CT due to neoadjuvant chemotherapy (delCT-RS), demonstrating that these parameters are excellent indicators for evaluating survival [35].

Li et al. retrospectively assessed the prognostic value of delta radiomics using CT features in predicting the prognosis of stage IV gastric cancer patients treated with ICI therapy. Eight radiomic features were identified in intratumoral and peritumoral regions in arterial and venous phases on baseline CT and follow-up CT scans. From these radiomic features, based on baseline and first follow-up CT scans, the authors assigned a specific score to the lesions, enabling the prediction of patients' progression-free survival [36].

Wang et al. studied how to develop and validate a radiomic model to assess Tumor Regression Grade (TRG) for patients with locally advanced gastric tumors after neoadjuvant chemotherapy. A total of 103 patients were retrospectively recruited and divided into two cohorts. Up to six radiomic features were finally selected for the neoadjuvant chemotherapy, post-neoadjuvant therapy, and delta feature sets. The delta model demonstrated the best performance in assessing TRG in both the training and validation cohorts. Therefore, the study highlighted that delta radiomics based on CT images could potentially serve as a biomarker for evaluating TRG and predicting patient prognosis [37].

#### Liver

The delta radiomic approach has been also tested for assessing treatment response and characterizing hepatocellular carcinoma (HCC) as well as hepatic metastases.

Aujay et al. evaluated the ability of radiomics to assess the response to transarterial radioembolization (TARE), enrolling 22 patients with HCC who underwent magnetic resonance imaging 4 weeks before and 4 weeks after treatment. By assessing long-run emphasis, minor axis length, surface area, and gray level nonuniformity on arterial phase images, they could identify responders and non-responders [38].

Ho et al. demonstrated the importance of the multifaceted radiomic association based on pre-treatment MRI, resulting from the combination of sequential transarterial chemoembolization (TACE) and stereotactic body radiotherapy (SBRT) associated with immunotherapy in patients with unresectable HCC. Retrospectively evaluating 26 patients, they showed the feasibility of identifying responsive patients, and thus candidates for treatment, through four radiomic characteristics: temporal change, intratumoral uniformity, radiomic features derived from the arterial phase (AP), and tumor morphology [39].

Similarly, Jin et al. assessed changes in texture characteristics induced by ablative SBRT in patients with HCC or cholangiocarcinoma. Delta radiomic features after a single dose of radiotherapy predicted local control in the studied patient cohort, allowing the identification of patients with radioresistant disease and providing physicians with the opportunity to modify patient management before standard retreatment at 3 months [40].

While the above-mentioned studies focused on predicting treatment response through the study of delta radiomic features, Han et al. retrospectively studied delta radiomics on contrast-enhanced hepatospecific MRI scans. They observed that the delta radiomic model can non-invasively predict glypican-3-positive HCC, providing valuable information for diagnosis and personalized treatment [41].

Liver metastases, especially from colorectal carcinoma, have been extensively studied in recent years using delta radiomic models, particularly to assess the degree of response in both pre and post-treatment settings.

Ye et al. studied the association of delta radiomic features based on CT with progression-free survival in patients with colorectal liver metastases undergoing chemotherapy. Through delta radiomic data, they evaluated the response to first-line oxaliplatin-based treatment of individual hepatic metastases, predicting patients who would develop resistance to treatment and avoiding unnecessary drug toxicity [42, 43].

Similarly, Su et al. assessed the efficacy and outcomes of treatment through a radiomic model based on magnetic resonance imaging. They enrolled 100 patients, categorized as responders and non-responders, and extracted data from images before and after treatment. They found the advantages of the radiomic model based on the difference in radiomic features of images in predicting treatment response, thereby improving decision-making and clinical outcomes [44].

#### Pancreas

Only two studies have utilized delta radiomic models in the pancreas in recent years, and both aimed at evaluating treatment response.

Simpson et al. extracted delta radiomics from low-field magnetic resonance imaging in 30 patients with pancreatic carcinoma. The study aimed to predict treatment response using images treated with magnetic resonance imaging (MRI)-guided ablative stereotactic radiotherapy [45]. Similarly, Tomaszewski et al. evaluated 26 patients with pancreatic carcinoma undergoing MRI-guided stereotactic radiotherapy to predict treatment response through delta radiomic analyses [46].

#### Rectal cancer

The assessment of delta radiomic data can serve as a biomarker for various contexts also in rectal cancer, especially in evaluating the response to neoadjuvant chemoradiation treatment.

Wen et al. examined 126 patients with locally advanced rectal carcinoma (LARC) to assess the response to neoadjuvant chemoradiation treatment and avoid surgery. Prediction of treatment response was evaluated using pre- and post-therapy characteristics and delta radiomic features based on magnetic resonance imaging. The radiomic models were compared with the qualitative assessment of two radiologists. While the author urged caution regarding this evidence, it was demonstrated that radiomic models were more sensitive compared to individual radiologists' evaluations, as the latter tended to overestimate the disease [47].

In the THUNDER-2 study, Chiloiro et al. assessed as the use of delta radiomics and the introduction of an Early Regression Index (ERI) could predict the response to radiotherapy. Their goal was to evaluate the impact of increasing radiotherapy dose in low-response LARC patients, identified by ERI, calculated between 0.35 T MRI simulation imaging and mid treatment one (BED 22 Gy), with the aim of enhancing complete response and avoiding surgical treatment [48].

Subsequently, Chiloiro et al. evaluated treatment response in LARC patients undergoing neoadjuvant chemoradiation through delta radiomics applied to mesorectal features. Pre- and post-treatment magnetic resonances were analyzed and predictive models for complete pathological response were developed to integrate with ERI [49].

In addition to features extracted from the mesorectum, Chiloiro et al. developed an additional logistic regression model capable of predicting 2-year disease-free survival (2yDFS) through the delta radiomic approach. The study results suggested promising preliminary outcomes, especially in the predictive 2yDFS model, based on the variation in terms of the area/surface ratio between biologically effective doses (BED) at 54 Gy and simulations [50].

The same authors subsequently published a second article from the THUNDER-2 study, in which they continued this project, demonstrating the feasibility of the predictive model based on delta radiomics. The increase in dose up to 60.1 Gy is well-tolerated in LARC patients predicted as non-responders by ERI, confirming the safety of this approach, especially in terms of acute toxicity and treatment adherence [51].

Similarly, Wang et al. developed and validated delta radiomic models based on MRI to predict treatment response in LARC patients undergoing neoadjuvant chemoradiation, highlighting the potential in clinical application for predicting response and providing increasingly personalized therapies [52].

Nardone et al. evaluated the use of MRI in delta texture analysis (D-TA) in predicting the frequency of complete pathological responses and therefore the survival of LARC patients undergoing neoadjuvant chemoradiation and subsequent radical surgery. The results appeared particularly promising, supporting the hypothesis that D-TA may have significant predictive value in detecting the onset of complete response and/or in selecting patients who may benefit from radical surgery after chemoradiation treatment [53].

Peng et al. retrospectively studied the predictive efficacy of radiomic features of MRI at different points in neoadjuvant therapy in patients with rectal carcinoma who showed a complete response at the end of treatment. They developed and validated a spatiotemporal radiomic model (RSTM) using artificial intelligence. The RSTM demonstrated excellent predictive efficacy in complete response to neoadjuvant therapy, emerging as a potential clinical tool to assist in the management of patients with rectal cancer [54].

Lymph node involvement is still a highly debated topic. Zhu et al. aimed to study radiomic characteristics derived from T2-weighted images and ADC maps with magnetic resonance, before and after chemoradiation, in LARC patients to assess the status of metastatic lymph node disease, demonstrating that the radiomic model can predict distant lymph node disease [55].

#### Head and neck cancer

Several methods have been used to extrapolate radiomic delta models in predicting response to chemo-radiotherapy (CRT) treatments in head and neck (H&N) cancers.

Two studies used MRI to predict the possible response to induction chemotherapy (IC).

Xi et al. [56] analyzing pre and post IC MRI in primary nasopharyngeal carcinoma (NPC) selected 12 subsets from the radiomic delta model that could predict response to medical treatment.

Similarly, Corino et al. [57] found improved delta radiomic information from T2 images and ADC maps in predicting response to IC in sinonasal carcinoma.

Other authors have focused on cone-beam computed tomography (CBCT) analysis by calculating a delta of values between the first and last sessions of an RT treatment.

Trying to predict possible disease progression, Sellami et al. [58] showed seven radiomic signatures; among them only "coarseness" was shown to have a delta radiomic model effective in predicting tumor development.

Similarly, Morgan et al. obtained a model incorporating both clinical and radiomic features that succeeds in predicting local failure in CRT treatments of H&N cancers by analyzing a delta from the CBCT of the first and 21 sessions of RT [59].

This strategy can also be used to obtain data that can predict possible toxicity from CRT treatment; in fact, Abdollahi et al. combined radiomic parameters obtained from CBCT with dosimetric features from the parotid dose distribution to develop models for predicting xerostomia [60].

Jeong Kim et al. researched radiomic parameters from PET/CT scans pre and post RT treatment in patient with NPC, through delta radiomic showed a model capable in predicting PFS and OS [61].

#### Breast cancer

In breast cancers, two studies have focused on the use of MRI in the study of response in neoadjuvant chemotherapy (NAC) treatments.

Liu et al. researched the radiomic elements of pre-treatment MRIs and after the first cycle of NAC for possible response factors in predicting complete response at the axillary lymph node.

The results obtained are three delta radiomic models combined with clinical models capable of predicting the complete response at the axillary region [62].

Similarly, Guo et al. studied MRI pre- and post-first cycle of NAC, obtaining a computational model of early complete tumor response by dynamic contrast enhanced [63].

In the same setting of patients, Bhardwaj et al. focused on the study of radiomic features using ultrasound (US). By performing a delta of the values obtained analyzing US scans pre-NAC and at the fourth week of treatment, they determined features capable of predicting possible locoregional recurrence [64].

Using US, Yang et al. focused on looking for factors that predicted response to NAC in the same group of patients.

By analyzing pre-treatment images and after the second cycle of chemotherapy, they obtained a nomogram incorporating radiomic features and Ki-67 expression; this showed excellent prediction of response to neoadjuvant treatment [65].

One study focused on the use of delta radiomic of CT images in patients with metastatic breast cancer.

Khorrami et al. searched for radiomic patterns that could predict the response of liver metastases to systemic treatment with Cyclin-dependent kinase 4/6 inhibitors (CDK4/6i) by studying CT images pretreatment and after a few cycles of chemotherapy. Image comparison found a different change in intratumoral Haralick entropy between responding and nonresponding patients; an increase in this entropy was associated with higher nonresponse and poor OS, demonstrating that this value can be used in predicting patient outcome [66].

#### Other diseases

Some studies have focused on other more uncommon oncologic diseases such as cervical cancers, renal cancer, lymphomas and melanoma. While other authors have evaluated the use of delta radiomic not related to oncologic response outcomes but to other clinical conditions.

Small et al. developed a radiomic delta model based on the use of MRI in cervical cancer undergoing CRT followed by brachytherapy.

By analyzing MRI images of the first and last brachytherapy fraction, they obtained two delta radiomic features combinations associated with the prediction of PFS [67]. Instead, Esposito et al. studied if the use of PET/CT could predict response to chemotherapy in patients with follicular lymphoma analyzing pre- and post-therapy images in patients receiving two different types of chemotherapy. Although limited by a short follow-up period, they revealed a radiomic feature that could predict treatment response: patients with an initially low bone mineral density correlated with more aggressive disease with a shorter PFS [68].

In patients with metastatic melanoma, Guerrisi et al. tried to predict response to treatment with Nivolumab by studying CTs at baseline and after the first cycle of therapy. By analyzing the images, they found various promising radiomic features, among them the radiomic delta analysis showed that the greater variation in the percentage of intralesional entropy was related to better OS and PFS [69].

Miao et al. attempted to build an effective prediction model for neoadjuvant RT and targeted therapy based on whole-tumor texture analysis using multi-sequence MRI for patients with soft tissue sarcoma (STS) [70]. Their multi-sequence MRI whole-tumor texture analysis can effectively predict pCR status after neoadjuvant RT and targeted therapy in STS patients, outperforming RECIST criteria and standard AJCC staging.

As for non-oncological indications, some experiences have been published on the vascular system.

Li et al. investigated the use of delta radiomic in predicting treatment response in Moya Moya disease by studying pre- and post-revascularization CT scans; the result showed delta radiomic models based on "time to drain" with the potential for identification of collateral vessel formation after treatment [71]. Meanwhile, Gerwing et al. focused on studying delta radiomic factors in the possible prediction of response in venous malformations following sclerotherapy by analyzing MRI before and after therapy. Six distinct features were identified in this study that show promise in predicting the outcome of sclerotherapy [72].

Both studies highlight the significance of implementing non-oncology delta radiomics trials to further explore this area.

### Clinical trials

Several clinical trials are currently ongoing to investigate the role of delta radiomics within the diagnostic and therapeutic pathway of oncological and other diseases (Table 2).

**Table 2:** Characteristics of the clinical trials included in the meta-analysis

NCT number	Disease	Imaging	Methods	Endpoint	Location
NCT05466760	Breast	PET/MR	Prospective, Cohort study. Association of imaging data pre and post NAC with molecular subtypes	Prediction of treatment response	Taipei Veterans General Hospital, Taiwan
NCT04815694	Rectal	MRgRT	Interventional Single Group Assignment. Evaluation of RT response during treatment: if ERI > 13.1 dose boost on residual disease	Complete response validation of delta radiomics MRgRT	Fondazione Policlinico Universitario Agostino Gemelli IRCCS, Rome, Italy
NCT05465512	Gastric	CT	Prospective, Cohort study. Images before and after NAC were used to construct a deep learning-based radiomics signature to predict the efficacy of treatment	The predictive performance of imaging after neoadjuvant chemotherapy	Fujian Medical University, Fujian, China
NCT03958669	HCC	MRI/CT	Prospective, Cohort study. Molecular and image fingerprints are compared pre and post TKI treatment	Prediction for treatment outcome	University Hospital Tübingen, Tübingen, Germany
NCT05296434	Liver function	MRI	Prospective, Case–Control. Delta Radiomics Based on Gd-EOB-DTPA-enhanced MRI	Quantitative evaluation of liver function	Zhujiang Hospital, Guangdong, China

Abbreviations: NAC: Neoadjuvant chemotherapy, MRgRT: MRI guided Radiotherapy, ERI: Early tumor regression index, HCC: Hepatocellular carcinoma, TKI: Tyrosine kinase inhibitors, Gd-EOB-DTPA: Gadolinium ethoxybenzyl-diethylenetriaminepentaacetic acid

It is noticeable how certain studies aim to augment the delta radiomics algorithm with molecular/histological characteristics, supporting the theory that genomic knowledge of a condition can aid in treatment selection and prediction.

Additionally, the delta radiomics approach in adjusting the total RT dose during therapy upon early response proves to be interesting, paving the way toward innovative dose optimization paradigms.

Equally important is the role of delta radiomics in non-oncological contexts, providing additional insights into adverse events and supporting prevention strategies.

Clinical trials investigating delta radiomics in oncology and other fields hold the potential to revolutionize diagnostic and therapeutic approaches, offering personalized medicine to improve patient outcomes. As this research progresses, the integration of advanced imaging techniques with molecular data analysis is destined to reshape the medical decision-making process and ultimately advance precision medicine in many areas.

## Discussion

The aim of this systematic review is to assess the state of the art of the different delta radiomics approaches published in the scientific literature in the 2 years since our previous analysis and to evaluate the quality of the studies using the RQS and QUADAS-2.

Since our last work, there has been a significant increase in studies that focus on delta radiomics and MRI [73], especially for prostate cancer [74–77], gastrointestinal cancer [78–82], bone metastases [83, 84] and head and neck cancers [85–92].

This increase in research indicates a growing recognition of the potential benefits and nuances offered by this imaging modality. The literature expansion highlights a shift toward using advanced imaging techniques, such as MRI, in combination with delta radiomics.

The evaluation of the studies showed differences when comparing the QUADAS criteria and RQS scores.

Regarding QUADAS-2, 39.6% of the studies were classified as having a high or unclear risk of patients selection bias due to the ambiguity of the inclusion criteria. Regarding the index test, 58.6% of the studies were classified as high or unclear.

The risk of bias due to reference standard, flow and timing was classified as high or unclear in 36 (62.1%) and 30 (51.7%) studies.

If, on the other hand, we consider the applicability of QUADAS-2, it was also unclear or highly questionable in the areas of reference standard (20 studies, 34.5%), index test 23 studies, 49.1%), and patient selection (25 studies, 43.1%).

The median RQS of all studies was 30.5% (mean 31% ± 17%), with six studies (10.3%) having a quality score below 10% and 21 studies (36.2%) below 25%.

Eighteen studies did not perform any type of validation (31%), while 29 performed internal validation (50%) and 11 performed external validation (18%); these data are very similar to the ones reported in the previous review.

If we compare the data of our last review, we can see an improvement in the QUADAS scores in terms of high or unclear risk of risk bias in patient selection (60% vs. 39.6%), a small improvement in the data concerning the Index test (high risk 62% vs. 58, 6%) while a better value can be seen when analyzing the data concerning the unknown risk in workflow and timing (75% vs. 51.7%) while there is a slight decrease in the high risk of bias in timing and workflow (50% vs. 62.1%).

Additionally, if we compare the applicability of QUADAS-2, we find a slight worsening of the data in terms of the reference standard (60% vs. 34.5%), in the Index test (77.1% vs. 34.5%) and in the patient selection (70.8% vs. 43.1%).

The study quality showed a significant improvement when comparing the RQS values of the current and previous studies. The current median was 30.5%, compared to 25% in the previous studies [13].

It is important to highlight that in recent years, various approaches have been implemented to enhance the quality of radiomics studies. These include the use of checklists for both radiomics and artificial intelligence [93, 94], the standardization of image biomarkers [95], and tools designed to assess the risk of bias and the applicability of radiomics studies [96].

Nevertheless, delta radiomics remains a promising surrogate biomarker despite the described translational difficulties. Its application can simplify the evaluation of different therapeutic strategies, particularly in contexts where the desired endpoint is immediate and pragmatic. For example, in neoadjuvant strategies, where disease response is a crucial indicator of treatment efficacy.

This finding is particularly relevant given that cancer care requires timely and adaptive decision making, where the availability of a reliable biomarker can significantly guide clinical choices.

Interestingly, over a 2-year period, 58 articles were selected but only five ongoing clinical studies were found. This raises concerns about potential obstacles to the effective translational applications of delta radiomics in clinical practice. The limited number of active studies indicates a gap between theoretical advances in the literature and practical implementation in real world practice settings. We believe that having more clinical trials is essential to validate the reliability and applicability of delta radiomics approaches.

Despite having followed the most appropriate methodological approaches, the most impactful limitation of this systematic review is that the reported results were not quantified in a standardized approach. The considerable heterogeneity found in the methods and technical designs of the studies makes it difficult to directly compare and unambiguously evaluate the collected data, providing the reader with generalizable conclusions.

## Conclusions

Delta radiomics has shown promise in various clinical endpoints in oncology. These include differential diagnosis, prognosis, prediction of treatment response, and evaluation of adverse events. The studies included in this review suggest a positive trend in methodological rigor, transparency, and overall research quality in comparison to the previous systematic review [13]. However, the analysis reveals that a non-negligible percentage of the analyzed studies still exhibit a high risk of bias or low quality, which needs to be more directly addressed.

In conclusion, further research is necessary to clinically validate delta radiomics applications. Future investigations should include more prospective and multicenter studies to explore this innovative approach thoroughly and ensure greater reliability and robustness of the results obtained.

### Supplementary Information

Below is the link to the electronic supplementary material.

Supplementary file1 (PNG 198 KB)
