# Supplementary figures and images for: Delta radiomics: an updated systematic review
Source: Radiol Med. 2024 Jul 17;129(8):1197–214. doi: 10.1007/s11547-024-01853-4 (PMC11322237; doi:10.1007/s11547-024-01853-4)

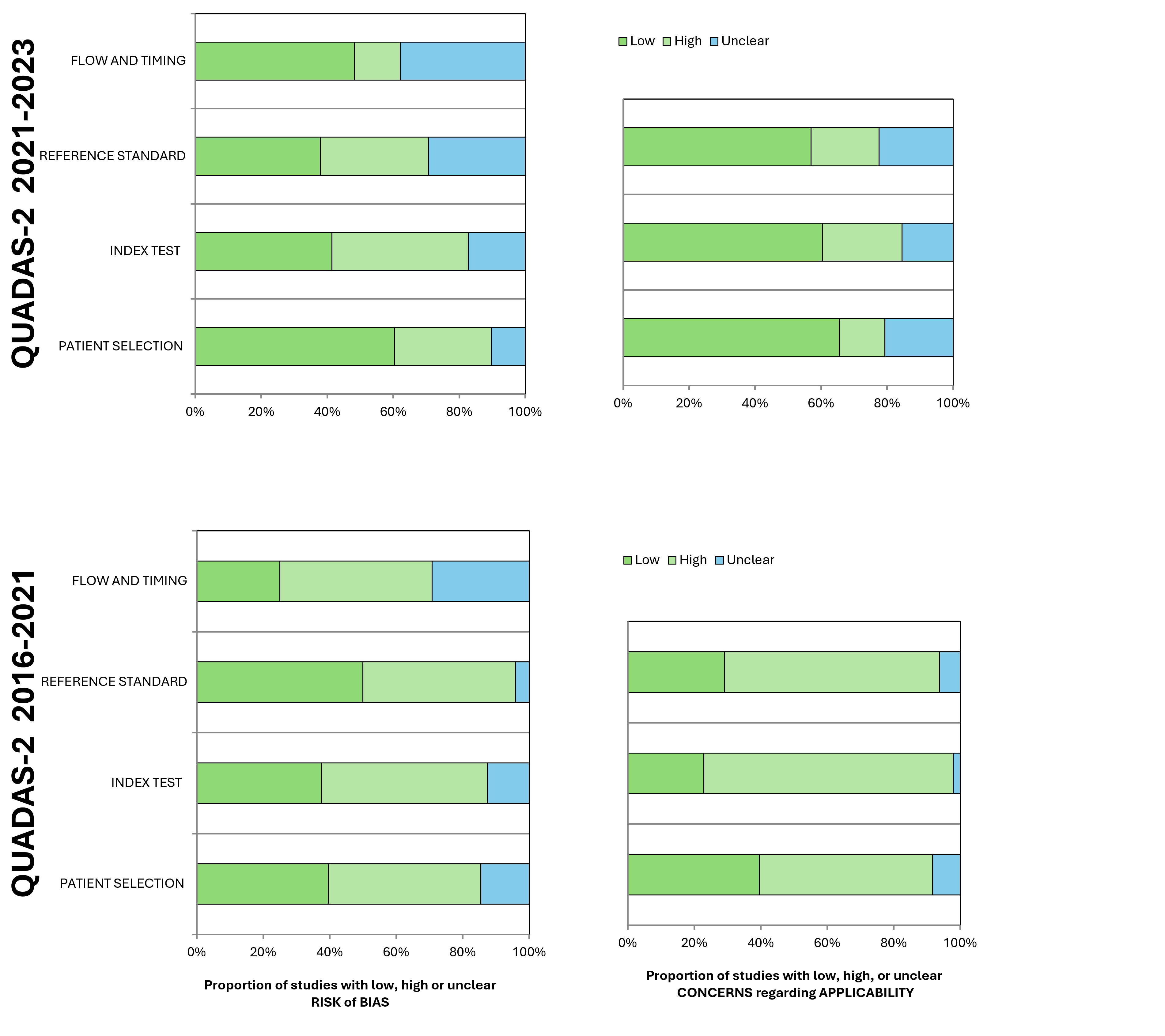

Supplement: Supplementary file 1 — Supplementary file1 (PNG 198 KB) [file 11547_2024_1853_MOESM1_ESM.png]
